# Incidence and risk factors for acute kidney injury after traumatic hemorrhagic shock: A 10-year retrospective cohort study

**DOI:** 10.1007/s40620-024-02035-1

**Published:** 2024-08-22

**Authors:** Xiujuan Zhao, Haiyan Xue, Chun Fu, Shu Li, Zhenzhou Wang, Ziyan Xiao, Jingjing Ye, Jie Cai, Yucun Yang, Qiong Zhao, Fengxue Zhu, Tianbing Wang, Wei Huang

**Affiliations:** 1 Department of Critical Care Medicine, Peking University People’s Hospital, Beijing, 100044 China; 2 Trauma Medicine Center, Peking University People’s Hospital, Beijing, 100044 China; 3 Key Laboratory of Trauma and Neural Regeneration (Peking University), Ministry of Education, Beijing, 100044 China; 4 National Center for Trauma Medicine of China, Beijing, 100044 China; 5 Department of Critical Care Medicine, Shenzhen Nanshan People’s Hospital, Shenzhen, 518052 China; 6 Department of Internal Medicine, Wudi Hospital of Traditional Chinese Medicine, Binzhou, 251900 China; 7 Emergency Department, First People’s Hospital of Jinghong, Xishuangbanna, 666100 China

**Keywords:** Trauma, Hemorrhage shock, Acute kidney injury, Risk factors

## Abstract

**Background:**

Acute kidney injury (AKI) is a common complication of traumatic hemorrhagic shock. The risk factors for AKI after traumatic hemorrhagic shock remain unclear. The aim of this study was to investigate the risk factors for AKI after traumatic hemorrhagic shock.

**Methods:**

This was a ten-year retrospective cohort study of patients who experienced traumatic hemorrhagic shock between January 2013 and April 2023. Patient characteristics and clinical data were recorded for 417 patients. The outcome was the occurrence of AKI, defined as a serum creatinine increase of ≥ 0.3 mg/dL (≥ 26.5 μmol/L) within 48 h, or an increase to 1.5 times the baseline, or a urine volume of < 0.5 mL/(kg h.). Risk factors for AKI were tested by logistic regression models.

**Results:**

The incidence of AKI after traumatic hemorrhagic shock was 29.3% (122/417 patients). Multivariable analysis revealed that the independent risk factors for AKI included age (OR, 1.048; 95% CI, 1.022–1.074; *p* < 0.001), B-type natriuretic peptide (OR, 1.002; 95% CI, 1.000–1.004; *p* = 0.041), sepsis (OR, 4.536; 95% CI, 1.651–12.462; *p* = 0.030) and acute myocardial injury (OR, 2.745; 95% CI, 1.027–7.342; *p* = 0.044). Road traffic accidents (OR, 0.202; 95% CI, 0.076–0.541; *p* = 0.001), mean arterial pressure (OR, 0.972; 95% CI, 0.950–0.995; *p* = 0.017), and base excess (OR, 0.842; 95% CI, 0.764–0.929; *p* = 0.001) were negatively correlated with AKI. The area under the receiver operating characteristic (ROC) curve for prediction by this model was 0.85 (95% CI, 0.81–0.90).

**Conclusion:**

The incidence of AKI after traumatic hemorrhagic shock was 29.3% in our series. Indicators of blood perfusion, sepsis and acute myocardial injury may be independent risk factors for AKI after traumatic hemorrhagic shock. Early detection and effective intervention on these risk factors could reduce the occurrence of AKI and improve outcomes.

**Graphical abstract:**

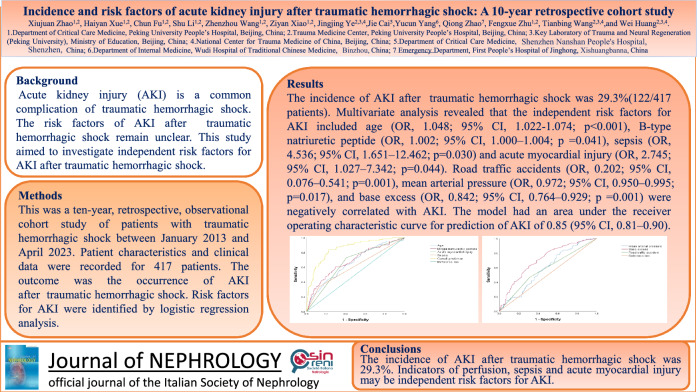

**Supplementary Information:**

The online version contains supplementary material available at 10.1007/s40620-024-02035-1.

## Introduction

Traumatic hemorrhagic shock is a condition in which massive bleeding caused by external or internal injury leads to reduced circulating blood volume, inadequate oxygen supply, and shock [1]. During hemorrhagic shock, the body experiences widespread tissue ischemia and cellular hypoxia, particularly in the kidneys [1]. A decrease in renal perfusion pressure can cause renal ischemia, leading to acute kidney injury (AKI) [2]. A multicenter cohort study showed that the incidence of AKI in patients with traumatic hemorrhagic shock reached 42.5%, significantly affecting the overall prognosis of trauma patients [3].

Acute kidney injury is a clinical syndrome characterized by rapid loss of renal function. It has various pathological mechanisms, such as acute tubular necrosis, acute interstitial nephritis, and decreased renal perfusion pressure [4]. In traumatic hemorrhagic shock, reduced systemic blood volume, renal hypoxia, and decreased renal perfusion pressure can result in insufficient renal blood flow, leading to AKI [5]. Additionally, hemorrhagic shock can cause a decrease in the glomerular filtration rate, an increase in blood lactate levels, an inflammatory response, and injury to renal tubular epithelial cells, contributing to the occurrence and progression of AKI [5].

The occurrence of AKI in patients with traumatic hemorrhagic shock is influenced by various factors, including the severity of the injury, the individual's baseline health status, the presence of complications, and the timeliness and effectiveness of treatment measures [6]. Early identification of, and active intervention in, the risk factors for AKI in patients with traumatic hemorrhagic shock, such as maintaining adequate blood circulation, avoiding nephrotoxic drugs, and following appropriate fluid management principles, are crucial for the prevention and reduction of AKI [7].

The early identification of risk factors could be important for preventing or alleviating AKI severity. Therefore, this study was aimed to determine the clinical characteristics and risk factors for AKI caused by traumatic hemorrhagic shock to provide a theoretical basis for identifying prevention strategies and improving clinical outcomes.

## Methods

### Study Design

This retrospective observational cohort study included all adult patients who were diagnosed with traumatic hemorrhagic shock at a tertiary teaching hospital between January 2013 and April 2023. Ethical approval was obtained from the Peking University People’s Hospital Medical Ethics Board (2020PHB258-01). This study was conducted according to the Strengthening the Reporting of Observational Studies in Epidemiology (STROBE) statement and the Declaration of Helsinki [8].

### Patients

The inclusion criteria were as follows: patients aged ≥ 18 years, patients who had experienced traumatic hemorrhagic shock, and patients with an expected length of hospital stay > 72 h. Traumatic hemorrhagic shock was defined as meeting the following criteria at admission (a,b,c or a,b,d): a, obvious bleeding caused by trauma (estimated to be greater than 1200 ml); b) hemoglobin < 100 g/L or > 30 g/L and lower than that before trauma; c, systolic blood pressure < 90 mmHg (or shock index [heart rate/systolic blood pressure] of > 1) on three consecutive measurements; and d, serum lactate > 2 mmol/L [9]. The exclusion criteria were pregnant or lactating women, patients with shock due to other causes, non traumatic patients, patients expected to die within 72 h of admission due to fatal trauma, patients with renal contusions or lacerations, patients with chronic kidney disease (CKD) stage 4–5 (estimated glomerular filtration rate [eGFR] < 30 mL/min per 1.73 m^2^, chronic dialysis, or prior kidney transplant), patients without baseline creatinine, and patients with incomplete data.

### Data collection

The data were collected consecutively and retrospectively. All data were obtained from the Trauma-Specific Database of the hospital, a real-world clinical database from 2012 to April 2023 that contains medical records on > 23,000 trauma cases. Training doctors and research nurses completed the data collection; they were unaware of the study and did not participate in the management or care of the patients. AKI and its clinical characteristics were documented. The data quality was assessed by reviewing a random sample of 10% of the data.

### Potential confounders and risk factors

Clinically relevant variables within 72 h after trauma included demographic characteristics, comorbidities (diabetes mellitus, hypertension, coronary artery disease, cerebral hemorrhage, and CKD), main bleeding site (thoracic, abdominal, pelvic, limbs, and others), cause of trauma (falling from a height, road traffic accident, falling from a standing position, and others), worsened mean arterial pressure and heart rate, and worsened laboratory data. Organ dysfunction (acute myocardial injury, acute respiratory distress syndrome (ARDS), and acute liver injury), injury severity score (ISS), and Acute Physiology and Chronic Health Evaluation II (APACHE II) score within 24 h after trauma were evaluated. The time from trauma to admission was recorded, and sepsis that occurred within 72 h after trauma was evaluated. The diagnosis and treatment measures included contrast agents, the amount of red blood cells transfused, and vasopressors (including norepinephrine, dopamine, adrenaline, and vasopressin) within 72 h after trauma.

Acute myocardial injury was defined as a cardiac troponin I (cTNI) concentration above the upper reference limit (URL) of the 99th percentile. Injury was considered acute if there was an increase or decrease in cTNI values [10]. The cTNI level was assessed using a high-sensitivity troponin I assay on a DxI800 (Beckman Coulter, Brea, CA, USA), and the 99th percentile for this test was 0.034 ng/mL. Acute respiratory distress syndrome was defined according to the Berlin definition [11]. Acute liver injury was defined as a serum total bilirubin level > 34.2 μmol/L or serum alanine aminotransferase and aspartate aminotransferase levels more than twice as high as the normal values, two liver function tests with abnormal results after injury, and no history of acute or chronic hepatitis or liver cirrhosis [12]. Sepsis was defined according to the third international consensus definitions for sepsis and septic shock [13].

### Outcome

The primary outcome was the occurrence of AKI within 7 days of traumatic hemorrhagic shock. AKI was defined as a serum creatinine increase of ≥ 0.3 mg/dL (≥ 26.5 μmol/L) within 48 h, or an increase to 1.5 times the baseline, or a urine volume of < 0.5 mL/(kg·h) for at least 6 h [14]. The baseline creatinine level was defined as the mean creatinine level in the 3 months before admission. AKI stage was determined according to the Kidney Disease Improving Global Outcomes (KDIGO) clinical practice guidelines [14].

### Statistical analysis

The sample size for the logistic regression model was calculated using NCSS-PASS 11 sample size estimation software. This study focused primarily on the relationship between AKI after traumatic hemorrhagic shock and acute myocardial injury. The incidence of AKI was considered as approximately 50%. We calculated a sample size large enough to detect an odds ratio (OR) of 3.0, with 85% power at the 0.1 significance level, using a two-sided test. After the calculation, the required sample size was 403.

Quantitative and qualitative variables are presented as the mean ± standard deviation (SD), median (25th, 75th percentile), and number and percentage. Continuous variables were compared using Student’s *t* test or the nonparametric Mann–Whitney *U* test, and categorical variables were compared using Pearson’s χ2 test or Fisher’s exact test. Collinearity diagnostics were performed for all risk factors. A logistic regression model was built using a backward stepwise selection procedure to identify the independent risk factors for AKI after traumatic hemorrhagic shock. Adjusted ORs and their 95% confidence intervals (95% CIs) were calculated. We used the Hosmer–Lemeshow test to evaluate the calibration of the logistic regression model. We assessed the discrimination ability of the models using the C-statistic (area under the receiver operating characteristic curve). The calibration curve was drawn by performing internal validation with the bootstrap resampling method.

All *p* values were 2-tailed, and *p* < 0.05 was statistically significant. SPSS 25.0 for Windows (SPSS, Chicago, IL, USA) and R software (version 4.3.2) were used for the statistical analysis.

## Results

### Patient characteristics

**Fig.1:**
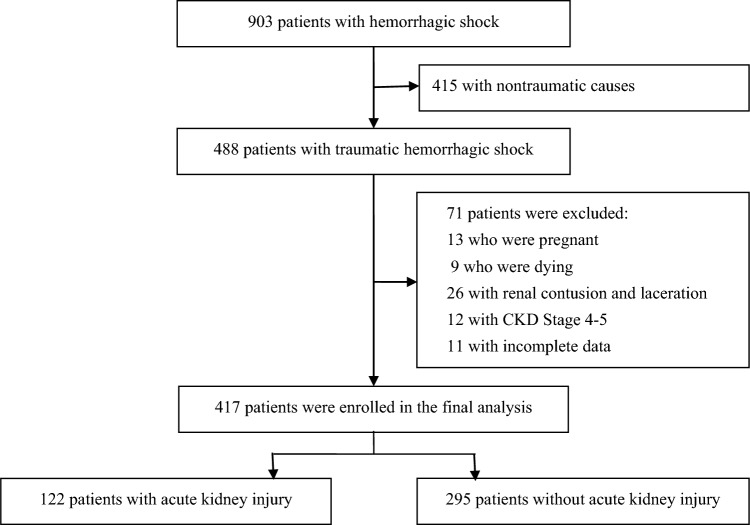
Patient flowchart

A total of 903 patients with hemorrhagic shock were referred to our institution, 488 of whom met the inclusion criteria. The data from 417 patients were analyzed. The patient selection process is illustrated in Fig. 1. The clinical characteristics of the patients are summarized in Table S1. AKI occurred in 122 (29.3%) of the 417 patients with traumatic hemorrhagic shock. Among the 122 patients with AKI, the time from trauma to AKI was 18.0(12.3–44.3) hours. Eighty (65.6%) patients were classified as stage 1, 24 (19.7%) as stage 2, 18 (14.7%) as stage 3, and 4 (3.3%) as requiring kidney replacement therapy in the hospital. Of the patients who received kidney replacement therapy, 2 survived and 2 died. Both surviving patients were free from kidney replacement therapy, but their renal function had not recovered. The mortality rate of patients with traumatic hemorrhagic shock and AKI was 28/122 (23.0%), while that of patients without AKI was 12/295 (4.1%).

### Risk factors for acute kidney injury after traumatic hemorrhagic shock

Table 1 compares the risk factors between patients with and without AKI. Patients with AKI were significantly older than non-AKI patients (68.0 (54.5–84.0) vs. 56.0(44.0–73.0) years, *p* < 0.001). Patients with AKI exhibited greater rates of hypertension and CKD (44.3% vs. 23.7%, *p* < 0.001 and 5.7% vs. 0.3%, *p* < 0.001, respectively). Road traffic accidents were more common in the non-AKI group (44.1% vs. 30.3%, *p* = 0.007). The mean arterial pressure was lower in the AKI group (66.3 (52.3–78.0) vs. 71.7 (61.3–86.8) mmHg, *p* = 0.003). Leukocyte count, serum creatinine, activated partial thromboplastin time, D-dimer, serum creatine phosphokinase, myoglobin, serum lactate, C-reactive protein, serum procalcitonin, cardiac troponin I, and B-type natriuretic peptide levels were all greater in the AKI group (all *p* < 0.05). Hemoglobin, fibrinogen, and base excess levels were lower in the AKI group (all *p* < 0.05). Acute myocardial injury and acute liver injury were more common in the AKI group (*p* < 0.05). Disease severity indices (derived from the injury severity score—ISS and APACHE II score) were positively correlated with AKI development (*p* < 0.05). The amount of red blood cells transfused was greater, the use of vasopressors was more common, and the incidence of sepsis was greater in the AKI group than in the non-AKI group (all *p* < 0.05).

**Table 1:** Comparison of risk factors between traumatic hemorrhagic shock patients with and without acute kidney injury

Variable	Without acute kidney injury *n* = 295	Acute kidney injury *n* = 122	Test value	*p* Value
Male (*n*, %)	163 (55.3)	70 (57.4)	0.158	0.691
Age [M (P_25_-P_75_)], years	56.0 (44.0–73.0)	68.0 (54.5–84.0)	4.845	< 0.001
Preadmission conditions (*n*, %)				
Coronary heart disease	14 (4.7)	7 (5.7)	0.178	0.673
Hypertension	70(23.7)	54 (44.3)	17.416	< 0.001
Diabetes mellitus	32 (10.8)	21 (17.2)	3.152	0.076
Cerebral hemorrhage	24 (8.1)	9 (7.4)	0.068	0.794
Chronic kidney disease	1(0.3)	7(5.7)	13.369	< 0.001
Causes of trauma (*n*, %)				
Falling from a height	47(15.9)	21 (17.2)	0.104	0.747
Road traffic accident	130 (44.1)	37(30.3)	6.786	0.009
Falling from a standing position	96 (32.5)	52 (42.6)	3.831	0.050
Others (crush, stab, animal bite)	22 (7.5)	12 (9.8)	0.652	0.419
Main bleeding site (*n*, %)				
Thoracic	57 (19.3)	21 (17.2)	0.252	0.615
Abdominal	38 (12.9)	15 (12.3)	0.027	0.870
Pelvic	37(12.5)	20(16.4)	1.085	0.298
Limbs	118 (40.0)	45 (36.9)	0.352	0.553
Others (blood vessels, skin, and soft tissue)	44 (14.9)	22 (18.0)	0.494	0.482
Mean arterial pressure [M (P_25_-P_75_)], mmHg	71.7 (61.3–86.8)	66.3 (52.3–78.0)	2.977	0.003
Heart rate (mean ± SD)*,* beats/minute	106.3 ± 22.4	108.5 ± 23.5	0.909	0.364
Time from trauma to admission[M (P_25_-P_75_)], hours	7.0(4.0–12.0)	7.0(4.5–12.0)	0.254	0.800
Laboratory tests				
Leukocyte count [M (P_25_-P_75_)], × 10^9^/L	11.0 (8.2–15.8)	12.7 (8.6–18.7)	2.233	0.026
Neutrophil count [M (P_25_-P_75_)], × 10^9^/L	9.6 (6.7–14.0)	11.0 (6.8–16.4)	1.556	0.120
Lymphocyte count[M (P_25_-P_75_)], × 10^9^/L	0.8(0.5–1.2)	0.8(0.5–1.3)	0.887	0.375
Hemoglobin (mean ± SD), g/L	98.0 ± 22.9	89.8 ± 26.2	3.192	0.002
Platelet count [M (P_25_-P_75_)]*,* × 10^9^/L	135.0 (81.0–175.0)	119.0 (68.0–173.0)	1.766	0.077
Serum creatinine [M (P_25_-P_75_)], μmol/L	63.0 (50.5–77.5)	126.0 (103.0–163.0)	7.074	< 0.001
Total bilirubin [M (P_25_-P_75_)], μmol/L	17.3 (11.2–26.2)	15.6 (10.0–23.9)	1.539	0.124
Activated partial thromboplastin time[M (P25-P75)], s	28.6 (26.8–31.3)	31.3 (28.1–41.4)	3.740	< 0.001
Fibrinogen [M (P25-P75)], mg/dL	199.0 (149.0–307.5)	167.0 (138.0–317.0)	1.999	0.046
D-dimer [M (P25-P75)], ng/mL	5291.0 (2700.0–13379.0)	8513.0 (2161.0–31875.0)	3.028	0.002
pO2/FiO2 ratio [M (P25-P75)], mmHg	337.3 (240.4–408.8)	318.0 (239.7–391.5)	0.490	0.624
Serum lactate [M (P25-P75)], mmol/L	2.6 (1.5–3.6)	3.0 (1.9–5.5)	3.403	0.001
Base excess [M (P25-P75)], mmol/L	− 3.0 (− 5.0–− 1.0)	-6.0 (-9.0–-3.0)	6.269	< 0.001
C-reactive protein [M (P25-P75)], mg/L	62.8 (34.0–87.8)	66.9 (25.3–119.7)	2.021	0.043
Serum procalcitonin [M (P25-P75)], ng/mL	0.9 (0.5–3.5)	3.0 (1.0–8.0)	5.862	< 0.001
cTNI[M (P25-P75)], ng/mL	0.03 (0.009–0.19)	0.14 (0.02–0.99)	5.856	< 0.001
B-type natriuretic peptide [M (P_25_-P_75_)], pg/mL	56.0 (26.0–159.0)	158.0 (56.0–446.0)	5.691	< 0.001
Serum creatine phosphokinase [M (P_25_-P_75_)], U/L	1271.0(645.0–3040.0)	1575.0(874.5—4259.5)	2.708	0.007
Myoglobin [M (P_25_-P_75_)], ng/ml	634.6(403.0–1198.8)	1341.9(572.5–3157.6)	6.469	< 0.001
Organ dysfunction (*n*, %)				
ARDS	73 (24.7)	35 (28.7)	0.813	0.367
Acute myocardial injury	110 (37.3)	87(71.3)	41.586	< 0.001
Acute liver injury	76 (25.8)	49 (40.2)	8.987	0.003
Sepsis (*n*, %)	32(10.8)	42(34.4)	33.611	< 0.001
ISS (mean ± SD)	23.2 ± 9.7	23.9 ± 10.6	2.003	0.046
APACHE II score (mean ± SD)	16.0 ± 4.5	20.8 ± 6.5	7.576	< 0.001
Amount of red blood cells transfused [M (P_25_-P_75_)], Units	10.0(8.0–16.0)	12.0(8.0–17.0)	2.967	0.003
Contrast agents (*n*, %)	129(43.7)	62(50.8)	2.029	0.154
Vasopressors (*n*, %)	106(35.9)	63(51.6)	9.416	0.002

*cTNI* cardiac troponin I, *ARDS* acute respiratory distress syndrome, *ISS* injury severity score, *APACHE II* Acute Physiology and Chronic Health Evaluation II, *M (P*_*25*_*-P*_*75*_*)* median (25th percentile, 75th percentile)

**Fig. 2:**
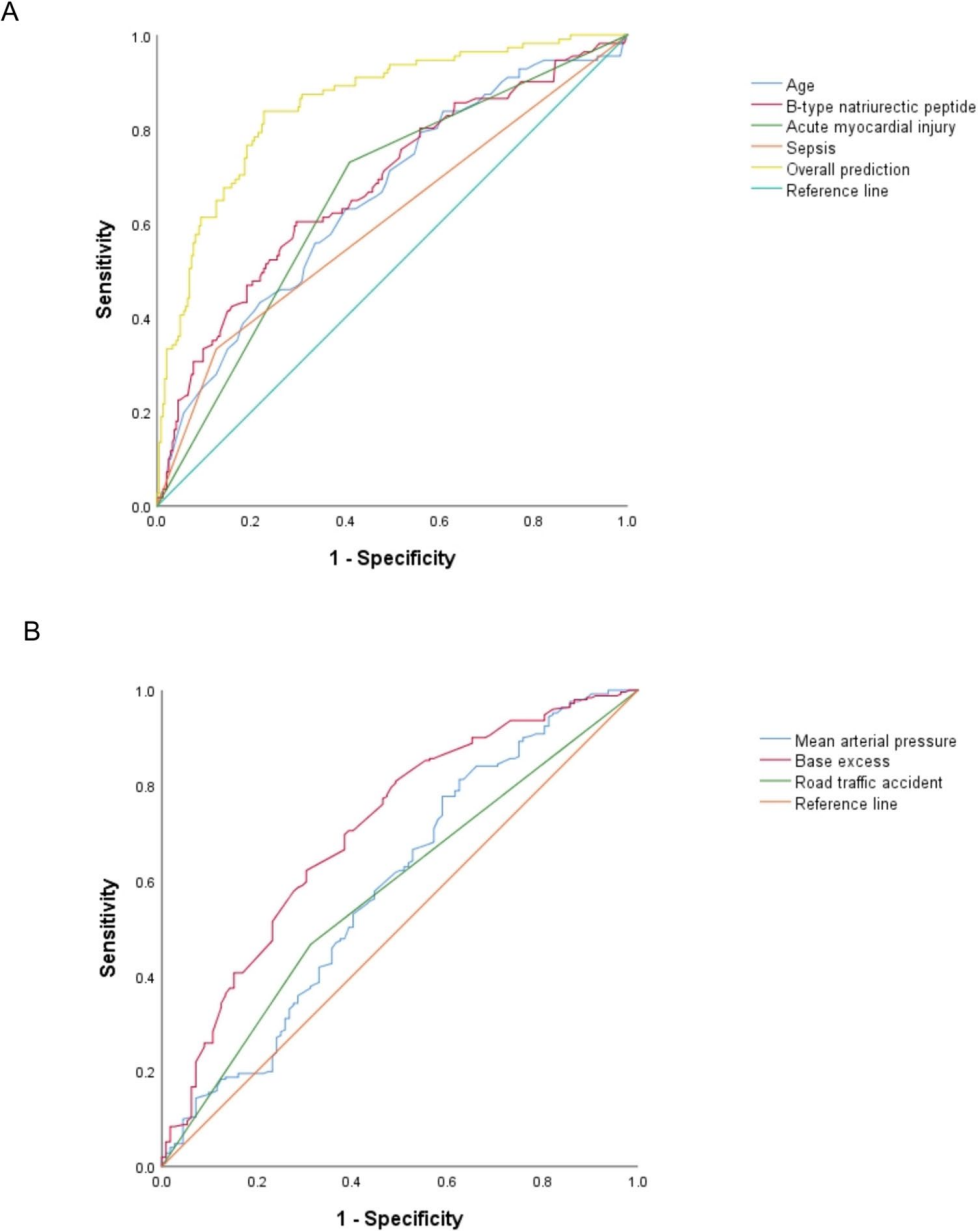
ROC curve of risk factors and the overall model for the prediction of acute kidney injury (**A**) and no acute kidney injury (**B**) after traumatic hemorrhagic shock

According to collinearity diagnostics, no variable had a tolerance of less than 0.1 or a variance inflation factor greater than 10. Therefore, there was no correlation between the variables. All statistically significant variables were included in a logistic regression model in which AKI after traumatic hemorrhagic shock was the dependent variable. The independent risk factors for AKI after traumatic hemorrhagic shock were age (OR, 1.048; 95% CI, 1.022–1.074; *p* < 0.001), B-type natriuretic peptide (OR, 1.002; 95% CI, 1.000–1.004; *p* = 0.041), sepsis (OR, 4.536; 95% CI, 1.651–12.462; *p* = 0.030) and acute myocardial injury (OR, 2.745; 95% CI, 1.027–7.342; *p* = 0.044). Road traffic accidents (OR, 0.202; 95% CI, 0.076–0.541; *p* = 0.001), mean arterial pressure (OR, 0.972; 95% CI, 0.950–0.995; *p* = 0.017), and base excess (OR, 0.842; 95% CI, 0.764–0.929; *p* = 0.001) were negatively correlated with AKI after traumatic hemorrhagic shock (Table 2). The AUCs for the prediction of AKI after traumatic hemorrhagic shock were 0.66 (95% CI, 0.60–0.72) for age, 0.58 (95% CI, 0.51–0.64) for road traffic accidents, 0.59 (95% CI, 0.52–0.66) for mean arterial pressure, 0.71 (95% CI, 0.65–0.77) for base excess, 0.68 (95% CI, 0.62–0.74) for B-type natriuretic peptide, 0.60 (95% CI, 0.54–0.67) for sepsis and 0.66 (95% CI, 0.60–0.72) for acute myocardial injury (Fig. 2). The AUC for the overall prediction with this logistic regression model was 0.85 (95% CI, 0.81–0.90) (Table S2) (Fig. 2), and the model showed good calibration (Hosmer–Lemeshow χ2 = 9.890; *p* = 0.273). The calibration plot showed good agreement between the actual observations and the predicted outcome (Figure S1).

**Table 2:** Univariate and multivariate logistic regression analysis of risk factors for acute kidney injury after traumatic hemorrhagic shock

Variable	Univariate analysis		Multivariate analysis	
	OR (95% CI)	*p*	OR (95% CI)	*p*
Age	1.028 (1.016–1.040)	< 0.001	1.048 (1.022–1.074)	< 0.001
Hypertension	2.602 (1.663–4.071)	< 0.001		
Chronic kidney disease	18.114 (2.204–148.837)	0.007		
Road traffic accident	0.562 (0.359–0.882)	0.012	0.202 (0.076–0.541)	0.001
Mean arterial pressure	0.984 (0.973–0.995)	0.005	0.972 (0.950–0.995)	0.017
Leukocyte count	1.042 (1.009–1.075)	0.011	1.053 (0.991–1.119)	0.097
Hemoglobin	0.986 (0.977–0.995)	0.002		
Activated partial thromboplastin time	1.051 (1.023–1.079)	< 0.001		
Fibrinogen	0.998 (0.997–1.000)	0.082		
D-Dimer	1.000 (1.000–1.000)	0.001		
Serum lactate	1.178 (1.093–1.270)	< 0.001		
Base excess	0.848 (0.801–0.897)	< 0.001	0.842 (0.764–0.929)	0.001
C-reactive protein	1.006 (1.002–1.009)	0.001		
Serum procalcitonin	1.061 (1.026–1.097)	< 0.001	1.045 (0.996–1.097)	0.072
cTNI	1.647 (1.215–2.231)	0.001		
B-type natriuretic peptide	1.003 (1.001–1.004)	< 0.001	1.002 (1.000–1.004)	0.041
Serum creatine phosphokinase	1.000 (1.000–1.000)	0.001		
Myoglobin	1.001 (1.000–1.001)	< 0.001		
Acute myocardial injury	4.327 (2.728–6.862)	< 0.001	2.745 (1.027–7.342)	0.044
Acute liver injury	1.970 (1.260–3.080)	0.003		
Sepsis	4.386 (2.597–7.408)	< 0.001	4.536 (1.651–12.462)	0.030
ISS	1.021 (1.000–1.043)	0.047		
APACHEII score	1.146 (1.100–1.194)	< 0.001		
Amount of red blood cells transfused	1.050 (1.020–1.081)	0.001		
Vasopressors	1.947 (1.268–2.989)	0.002		

*cTNI* cardiac troponin I, *ISS* injury severity score, APACHE II Acute Physiology and Chronic Health Evaluation II

## Discussion

In this study, the incidence of AKI in patients with traumatic hemorrhagic shock was 29.3%. Among the various factors investigated, age, B-type natriuretic peptide, sepsis, and acute myocardial injury were identified as independent risk factors. Road traffic accidents, mean arterial pressure, and base excess were negatively correlated with AKI.

The incidence of trauma-related AKI varies by the patient population, definition criteria, and study design. A retrospective study found that the incidence of trauma-related AKI was 13% but 42.5% in patients with hemorrhagic shock [3]. Several smaller studies have reported incidence rates of trauma-related AKI ranging from 20 to 50% [15]. In our study, among the 417 patients with traumatic hemorrhagic shock, 122 (29.3%) developed AKI, which is consistent with international reports. Other than hypovolemic shock, the major causes of trauma-related AKI include head trauma, multiple-organ dysfunction, and preexisting chronic diseases. A retrospective study from Italy [16] found that among 1617 adult patients with polytrauma, 62 (3.83%) patients required kidney replacement therapy. This finding was consistent with our results: only 4 (3.3%) of our AKI patients required kidney replacement therapy. A retrospective cohort study from Sweden [17] reported that kidney replacement therapy was required for 32% of patients with trauma in the intensive care unit, which was significantly higher than that in our study. Possible reasons include the ISS of 20 (14–29) in this study, which was lower than the ISS of 25 (17–34) in their study [17], and the timely treatment after trauma (time from trauma to admission, of 7 (4–12) hours) in this study.

We found that age was an independent risk factor for AKI after traumatic hemorrhagic shock, the AKI patients being older than the non-AKI patients. This may be due to a natural age-related decline in renal function, which increases the susceptibility of older individuals to kidney injury [18]. Furthermore, older individuals may have poorer physiological functions and weakened immune functions, increasing their vulnerability to kidney damage caused by imbalanced immune reactions after trauma [19]. Therefore, for older patients, close attention should be paid to their volume status, immune function, and renal baseline, a comprehensive analysis should be performed, and judgment should be made based on individual circumstances, with appropriate treatment and support measures implemented proactively.

Among the causes of trauma, road traffic accidents are less likely to result in AKI than falls from standing positions. Previous studies have reported a greater incidence of AKI in trauma patients involved in road traffic accidents [20]. However, this study presents findings that differ from previous ones, possibly due to several factors. One explanation for the contrasting results is the urban setting of this study, where road traffic accidents were more frequent. In urban areas, vehicle speeds are typically lower, and high-energy injuries are less common. These factors could contribute to a lower risk of AKI in road traffic accidents than in falls from a standing position. Furthermore, age and demographics play a role in the occurrence of AKI. In this study, road traffic accidents tended to occur more often in younger patients, with a median age of 55 years, whereas falls from a standing position typically occurred in older patients, with a median age of 81 years. Interestingly, the incidence of AKI increases significantly in older trauma patients after they experience hemorrhagic shock [4]. This disparity in age and associated factors might contribute to a lower likelihood of AKI caused by road traffic accidents than by falls from a standing position.

Hypoperfusion and hypoxia are common pathophysiological mechanisms of AKI [21]. Maintaining appropriate mean arterial pressure is crucial for stable renal perfusion. High arterial pressure can also increase the risk of tubular injury. Maintaining appropriate mean arterial pressure plays an important role in preserving renal function.

Sepsis is a significant risk factor for the occurrence of AKI in patients with traumatic hemorrhagic shock. In patients with sepsis, the activation of systemic inflammatory response syndrome and the massive release of inflammatory factors can cause microvascular damage to the kidneys and changes in hemodynamics [22]. These changes may lead to renal ischemia and oxidative stress, ultimately causing AKI [22]. The incidence of AKI after sepsis in China was reported to be as high as 47.1%, and is associated with  a higher risk of death [23]. Timely antibiotic therapy, appropriate fluid management, control of the source of infection, and stabilization of hemodynamics are key measures to reduce the risk of AKI in patients with sepsis [24]. In addition, for high-risk patients, early kidney replacement therapy may improve prognosis [24].

Our study also revealed that acute myocardial injury is another important risk factor for AKI caused by traumatic hemorrhagic shock. There are several possible reasons for this finding. First, acute myocardial injury can lead to reduced cardiac pumping, resulting in decreased blood pressure and unstable circulation. This circulatory failure can reduce renal blood perfusion and increase the risk of renal dysfunction [25]. Second, acute myocardial injury may lead to heart failure, and a decrease in cardiac pumping can lead to an excessive workload on the heart and increase the burden on the kidneys [25]. Consistent with this finding, we found that B-type natriuretic peptide (BNP) is an independent risk factor for traumatic AKI. Third, acute myocardial injury can intensify inflammatory reactions and systemic oxidative stress [26]. These inflammatory mediators and oxidative stress substances may affect the kidneys through the bloodstream, leading to reduced blood perfusion in the glomeruli and tubular damage, thereby exacerbating traumatic AKI [27]. In addition, the treatment of acute myocardial injury may require various interventions, including anticoagulation and the use of inotropic agents. The use of certain medications can increase the risk of kidney damage. Measures such as maintaining circulatory stability, appropriate fluid management, and reducing cardiac workload can help prevent and alleviate traumatic AKI [28].

Trauma-induced kidney injury can be either direct (kidney trauma) or indirect (such as a trauma-induced systemic inflammatory response causing kidney damage) [29]. Direct injuries include kidney rupture, bleeding, and injury to the kidney vessels. The post-traumatic inflammatory response and hypoxia can lead to ischemia‒reperfusion injury and cell damage in the kidneys [30]. Importantly, a patient may have both traumatic kidney injury and AKI for other reasons. Therefore, in the assessment and management process, multiple factors must be considered comprehensively, and detailed clinical evaluation and diagnosis must be performed.

This study has several limitations. First, it was a single-center retrospective cohort study. We did not perform simultaneous analyses at other centers for comparison, and there is relatively little available reference literature. Second, the entry of clinical data for all study subjects and evaluation of related scores were completed by multiple clinical staff members. To reduce or prevent human errors in the data collection process, we arranged for multiple individuals to regularly check, correct, and clean the data in the database. After excluding patients who did not meet the study criteria, the remaining sample was relatively small. Third, because AKI patients were older, had a greater comorbidity burden, and had more severe clinical conditions at admission, even after adjustment for these variables in the multivariate regression analysis, residual confounding was still possible. In the future, we will conduct larger, multidisciplinary, multicenter, prospective studies based on this clinical research to clarify the risk factors for AKI after traumatic hemorrhagic shock and to seek new biomarkers or clinical indicators, making a significant contribution to updating and improving the diagnosis and treatment strategies for AKI.

In conclusion, our study identified age, B-type natriuretic peptide, sepsis and acute myocardial injury as independent risk factors for AKI after traumatic hemorrhagic shock. Road traffic injuries, mean arterial pressure, and base excess were negatively correlated with AKI after traumatic hemorrhagic shock. Early detection and effective intervention for these risk factors can reduce the occurrence of AKI and improve patient outcomes. Large, prospective studies are needed to further clarify the epidemiology, prevention, diagnosis, treatment, and prognosis of AKI after traumatic hemorrhagic shock.

## Supplementary Information

Below is the link to the electronic supplementary material.

Supplementary file1 (DOCX 84 KB)

## Data Availability

The datasets generated and/or analyzed during the current study are not publicly available but are available from the corresponding author upon reasonable request.
