# Supplementary material for: Incidence and risk factors for acute kidney injury after traumatic hemorrhagic shock: A 10-year retrospective cohort study
Source: J Nephrol. 2024 Aug 22;37(8):2337–46. doi: 10.1007/s40620-024-02035-1 (PMC11649738; doi:10.1007/s40620-024-02035-1)
Supplement: Supplementary file 1 — Supplementary file1 (DOCX 84 KB) [file 40620_2024_2035_MOESM1_ESM.docx]

**Table S1** General and clinical characteristics of patients with traumatic hemorrhagic shock

| Characteristic | *n* = 417 |
| --- | --- |
| Male(*n*, %) | 233 (55.9) |
| Age [M (P_25_-P_75_)], years | 60.0 (47.5–78.0) |
| Preadmission conditions (*n*, %) |  |
| Coronary heart disease | 21(5.0) |
| Hypertension | 124 (29.7) |
| Diabetes mellitus | 53 (12.7) |
| Cerebral hemorrhage | 33 (7.9) |
| Chronic kidney disease | 8(1.9) |
| Causes of trauma (*n*, %) |  |
| Falling from a height | 68 (16.3) |
| Road traffic accident | 167 (40.0) |
| Falling from a standing position | 148 (35.5) |
| Others(crush, stab, animal bite) | 34 (8.2) |
| Main bleeding site (*n*, %) |  |
| Thoracic | 78 (18.7) |
| Abdominal | 53 (12.7) |
| Pelvic | 57 (13.7) |
| Limbs | 163 (39.1) |
| Others(blood vessels, skin, and soft tissue) | 66(15.8) |
| Time from trauma to admission [M (P_25_-P_75_)], hours | 7.0(4.5-12.0) |
| Time from trauma to AKI [M (P_25_-P_75_)], hours | 18.0(12.3-44.3) |
| Amount of red blood cells transfused [M (P_25_-P_75_)], Units | 12.0(8.0-17.0) |
| Contrast agents (*n*, %) | 191(45.8) |
| ISS [M (P_25_-P_75_)] | 20.0 (14.0–29.0) |
| APACHEII score [M (P_25_-P_75_)] | 17.0 (14.0–21.0) |
| Acute myocardial injury (*n*, %) | 197 (47.2) |
| Acute kidney injury (*n*, %) | 122 (29.3) |
| Sepsis (*n*, %) | 74(17.7) |
| In-hospital mortality (*n*, %) | 40 (9.6%) |
| Length of stay in hospital (days) | 17.0 (10.0–26.0) |
| Length of stay in ICU (days) | 11.0 (5.0–18.0) |

ISS, Injury Severity Score; APACHEII, Acute Physiology and Chronic Health Evaluation II; AKI, acute kidney injury; ICU, intensive care unit; M(P_25_-P_75_), median (25th percentile, 75th percentile).

**Table S2**AUCs for individual risk factors and for the overall model for the prediction of acute kidney injury after traumatic hemorrhagic shock

|  | AUC | |
| --- | --- | --- |
| Characteristic | OR (95% CI) | *p* |
| Age | 0.656(0.595–0.717) | <0.001 |
| Road traffic accident | 0.577(0.514–0.640) | 0.019 |
| Mean arterial pressure | 0.589(0.523–0.655) | 0.007 |
| Base excess | 0.706(0.647–0.765) | <0.001 |
| B-type natriuretic peptide | 0.681(0.620-0.742) | <0.001 |
| Acute myocardial injury | 0.660(0.600–0.721) | <0.001 |
| Sepsis | 0.604(0.538-0.670) | 0.002 |
| Overall | 0.854(0.811-0.896) | <0.001 |


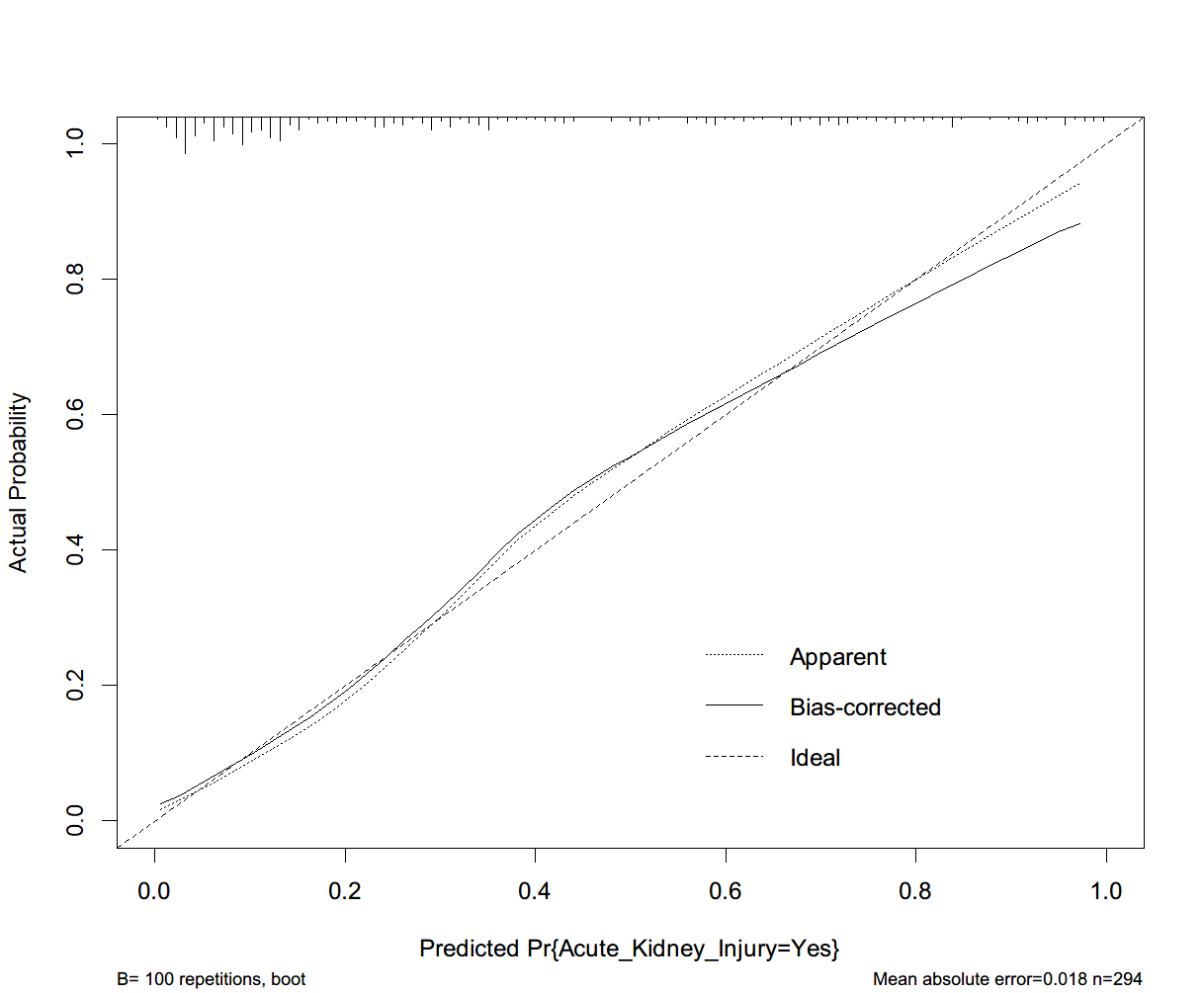


**Fig. S1**Calibration curve of the logistic model using bootstrapping
